# Assessment of epidermal growth factor receptor status in glioblastomas

**Published:** 2013

**Authors:** Hui-Jun Zhu, Mikako Ogawa, Yasuhiro Magata, Masahiko Hirata, Yoshiro Ohmomo, Hiroki Namba, Harumi Sakahara

**Affiliations:** 1 Department of Radiology, Hamamatsu University School of Medicine, Japan; 2 Medical Photonics Research Center, Hamamatsu University School of Medicine, Japan; 3 Osaka University of Pharmaceutical Sciences, Japan; 4 Department of Neurosurgery, Hamamatsu University School of Medicine, Japan

**Keywords:** [^125^I]PYK, Gefitinib, EGFR, Glioblastoma

## Abstract

**Objective(s):**

Our previous study showed that a newly designed tracer radioiodinated 6-(3-morpholinopropoxy)-7-ethoxy-4-(3’-iodophenoxy)quinazoline ([^125^I]PYK) is promising for the evaluation of the epidermal growth factor receptor (EGFR) status and prediction of gefitinib treatment of non-small cell lung cancer. EGFR is over-expressed and mutated also in glioblastoma. In the present study, the expressions and mutation of EGFR were tested with [^125^I] PYK in glioblastoma *in vitro* and *in vivo* to determine whether this could be used to predict the sensitivity of glioblastoma to gefitinib treatment.

**Methods:**

Glioblastoma cell lines with different expression of EGFR were tested. Growth inhibition of cell lines by gefitinib was assessed by the 3-(4, 5-dimethylthiazol-2-yl)-2, 5-diphenyltetrazolium bromide (MTT) colorimetric assay. Uptake levels of [^125^I]PYK were evaluated in cell lines *in vitro*. Tumor targeting of [^125^I]PYK was examined by a biodistribution study and imaging by single photon emission computed tomography (SPECT).

**Results:**

High concentrations of gefitinib were needed to suppress EGFR-mediated proliferation. The uptake of [^125^I] PYK in cell lines *in vitro* was low, and showed no correlation with EGFR expression or mutation status. Biodistribution study and SPECT imaging with [^125^I]PYK for xenografts showed no [^125^I]PYK uptake.

**Conclusion:**

The results showed prediction of gefitinib effectiveness was difficult in glioblastoma by [^125^I]PYK, which might be due to the complicated expression of EGFR status in glioblastoma. Thus, new tracers for sites downstream of the mutant EGFR should be investigated in further studies.

## Introduction

Glioblastoma multiforme (GBM) is the most common and highly aggressive malignant neoplasm of the central nervous system (CNS) (1). Despite the development of multi-treatment regimens, the treatment of glioblastomas remains difficult. Patients with GBM have a poor prognosis, with a median survival of approximately 12 months (2). The abnormal alteration of epidermal growth factor receptor (EGFR) genes is associated with the progression of GMB. About 40% of primary GBM show amplification of the EGFR gene and subsequent elevated levels of EGFR protein (3). About 63% to 75% of GBM with EGFR over-expression have been found with mutation of the EGFR genes. The aberrant EGFR alterations in GBM have been considered as novel targets for diagnostic, prognostic and therapeutic purposes (4).

Much effort has been expended in developing EGFR-targeted therapies. One promising approach is focused on epidermal growth factor receptor tyrosine kinase (EGFR TK) inhibitors. For example, geftinib and erlotinib could competitively bind to the adenosine triphosphate (ATP)-binding pocket in the kinase domain (5). Some preclinical studies have also shown promise of the EGFR TK inhibitor for GBM (6). However, the objective response rate for GBM patients treated with EGFR TK inhibitors is only 10 to 20 percent in clinical trials (7, 8). Therefore, it is important that a new approach to characterize EGFR expression and mutation in individual GBM patients is developed.

Quantitative non-invasive molecular imaging might aid in developing EGFR TK inhibitors and in patient selection for EGFR TK inhibitors. Some radiolabeled agents for molecular imaging have been successfully used for detecting EGFR expression in non-small cell lung carcinomas (NSCLC) (9). However, there is a lack of studies on the detection of mutant EGFR genes in GBM. In our previous study, an EGFR molecular imaging tracer radioiodinated 6-(3-morpholinopropoxy)-7-ethoxy-4-(3’-iodophenoxy) quinazoline ([^125^I]PYK) was developed to detect EGFR expression and mutation. It predicted whether gefitinib would be effective in NSCLC, and we showed previosuly that the target tumor could be clearly visualized by [^125^I]PYK-SPECT (10, 11). Now we present *in vivo* and *in vitro* studies of [^125^I]PYK aimed at detecting EGFR expression status in GBM.

## Materials and Methods

### 

#### Radiolabeling of PYK

[^125^I]NaI was obtained from MP Biomedicals Japan (Tokyo, Japan). [^125^I]PYK was synthesized according to the method previously reported using [^125^I]NaI (10). Briefly, aqueous hydrogen peroxide (10 μL, 30%) was added to a mixture of [^125^I]NaI (10 μL, 37.0 MBq, 74 TBq/mmol), 0.1 M HCl (25 μL) and the tributylstannyl precursor of PYK (0.01 mg in 10 μL ethanol). After 10 min reaction, [^125^I]PYK was purified by HPLC. Radiochemical yield was 97.5%, and radiochemical purity was more than 95%.

#### Cells

The human glioblastoma cell lines were kindly provided by Professor Motoo Nagane. U87MG, U87MG.ΔEGFR, U87MG.wtEGFR, and U87MG.DK used in this study were described previously (12). These cells showed the following different EGFR expressions: U87MG expresses a low amount of wild-type EGFR; U87MG.ΔEGFR overexpresses the type III deletion-mutant gene for EGFR; U87MG.DK expresses a kinase-deficient mutant of ΔEGFR; and U87MG.wtEGFR expresses exogenous wild type (wt) EGFR (12). Human epithelial carcinoma cell line A431 with a high expression of EGFR, was used as the positive control. Cell lines were routinely cultured in DMEM supplemented with 10% fetal bovine serum and were maintained at 37 °C in a humidified 95% air - 5% CO_2_ atmosphere.

#### Binding specificity of [^125^I]PYK to glioblastoma cells

Gefitinib was purchased from Cayman chemical (Michigan, USA). Competitive inhibition experiments were carried out in order to test the specificity of the compound in inhibiting EGFR. Cells were grown in 6-well plates and pretreated by 10 μM gefitinib for 10 min at 37 °C. Then 0.74 kBq [^125^I]PYK was added to the a cell suspension of 1.0×10^6^ cells in 1 mL PBS. The radioactivity incorporated in the cells was determined on a gamma counter.

#### Inhibition of tumor cell growth by gefitinib *in vitro*

Growth inhibition was assessed using 3-(4,5-dimethylthiazol-2-yl)-2, 5-diphenyltetrazolium bromide (MTT) colorimetric assay. Cells were seeded at 5000 per well in 96-well plates. Cells were treated with various concentrations of gefitinib (0.05-20 μmol/L) for 72 hr at 37 °C, followed by incubation for 2 hr at 37 °C with 10 mg/mL MTT. The results were determined by measuring the optical density of cell lysates at a wavelength of 570 nm. The IC_50_ values were defined as the concentration of gefitinib needed for a 50% reduction based on cell growth curves.

#### Xenografts model

Approximately 1×10^6^ glioblastoma cells in phosphate-buffered saline (PBS) were injected subcutaneously into the flanks of 4- to 6-wk-old female BALB/c nude mice. Tumor volumes were measured by the formula (length×width^2^) ×0.5.

#### Biodistribution study of [^125^I]PYK

Nude mice (n=4) with xenograft tumors received tail vein injections of 37 kBq of [^125I^]PYK in 0.1 mL of saline for biodistribution study. At 24 hr after injection, blood, lung, muscle, heart, spleen, stomach, small intestine, kidney, liver and tumor samples were collected and weighed, and radioactivity was assayed in a gamma counter. The results were calculated as percentages of injected dose (ID) per gram of tissue.

#### Imaging of nude mice with xenografts with SPECT

Nude mice (n=4) bearing U87MG. ΔEGFR xenograft underwent single photon emission computed tomography (SPECT) scans when the tumor volume reached 300–400 mm^3^. The mice were injected with 12.95 MBq of [^125^I]PYK and imaged on the Micro-PET/SPECT/CT scanner (Gamma Medica-Ideas, Inc., Northridge, CA). The scans were performed at 1, 3 and 24 hr after injection.

## Results

### 

#### Binding specificity of [^125^I]PYK to glioblastoma cells

**Figure 1:**
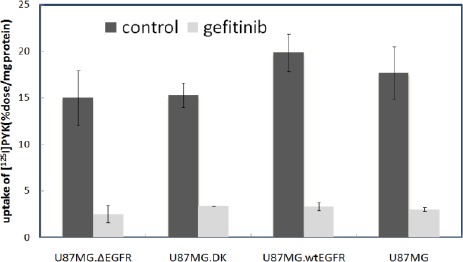
Decreased uptake of [^125^I]PYK by cultured cells after treatment with gefitinib, data represent mean ± SD, n =3.

The binding of [^125^I]PYK to glioblastoma cells was inhibited by pretreatment with gefitinib (Figure 1). The specific binding of [^125^I]PYK, however, was similar in all cell lines, and there was no correlation with EGFR expression and mutation.

#### Inhibition of tumor cell growth by gefitinib *in vitro*

MTT assay showed U87 cell lines had similar sensitivities to gefitinib in each other, but required higher doses of gefitinib compared with representative gefitinib-sensitive A431 cell lines (Table 1).

**Table 1:** The cell growth and EGFR TK activities inhibition of U87 cell lines by gefitinib

Cell lines	IC50(μM)
A431	0.31 ± 0.09
U87MG.⧍EGFR	15.3 ± 0.3
U87MG.DK	16.7 ± 1.9
U87MG.wtEGFR	26.7 ± 1.3
U87MG	14.2 ± 1.6

Cells were measured after a 72 hr incubation of gefitinib, assays for all cell lines were performed in triplicate

#### Biodistribution of [^125^I]PYK in nude mice

The biodistribution properties of [^125I^]PYK are shown in Table 2. The distributions of [^125^I]PYK in tumors and organs were evaluated at 24 hr post injection. Normal organ uptake of radioactivity was highest in colon. High tumor/muscle and tumor/blood ratios were observed in all cell lines. However, the accumulated radioactivity in the tumor was not high enough for *in vivo* tumor detection.

**Table 2:** Biodistribution of [^125^I]PYK in nude mice at 24h post-injection (%ID/g)^a^

Tissue	Xenograft of cell lines			
	U87MG.AEGFR	U87MG.DK	U87MG.wtEGFR	U87MG
Tumor	0.17 ± 0.06	0.24 ± 0.16	0.18 ± 0.07	0.15 ± 0.02
Blood	0.05 ± 0.01	0.05 ± 0.00	0.05 ± 0.01	0.05 ± 0.01
Pancreas	0.06 ± 0.03	0.20 ± 0.02	0.15 ± 0.04	0.21 ± 0.02
Spleen	0.12 ± 0.00	0.16 ± 0.01	0.13 ± 0.01	0.17 ± 0.03
Stomach	0.62 ± 0.56	0.38 ± 0.54	0.54 ± 0.47	0.64 ± 0.80
Small intestine	0.59 ± 0.28	0.87 ± 0.13	0.79 ± 0.52	1.01 ± 0.23
Colon	1.69 ± 0.41	5.32 ± 1.22	3.26 ± 1.49	5.57 ± 0.96
Liver	0.42 ± 0.14	0.47 ± 0.05	0.48 ± 0.12	0.55 ± 0.09
Kidney	0.30 ± 0.05	0.32 ± 0.03	0.25 ± 0.04	0.34 ± 0.02
Heart	0.09 ± 0.02	0.11 ± 0.01	0.08 ± 0.01	0.09 ± 0.01
Lung	0.17 ± 0.04	0.21 ± 0.08	0.16 ± 0.01	0.22 ± 0.03
Brain	0.03 ± 0.01	0.03 ± 0.00	0.02 ± 0.01	0.03 ± 0.01
Muscle	0.05 ± 0.01	0.08 ± 0.05	0.06 ± 0.02	0.07 ± 0.01
Bone	0.31 ± 0.07	0.32 ± 0.05	0.36 ± 0.15	0.37 ± 0.15
Uptake ratio ^b^				
Brain/Blood	0.64 ± 0.17	0.56 ± 0.03	0.48 ± 0.17	0.61 ± 0.13
Tumor/Muscle	3.78 ± 1.09	4.23 ± 3.47	3.29 ± 1.83	2.16 ± 0.33
Tumor/Blood	3.90 ± 1.67	4.71 ± 3.25	3.89 ± 1.67	2.95 ± 0.60

a The data are expressed as Mean % injection dose ± S.D. per gram, n=4

b Ratio of brain to blood or tumor-to-normal tissue.

#### In vivo SPET/CT Imaging with [^125^I]PYK

**Figure 2:**
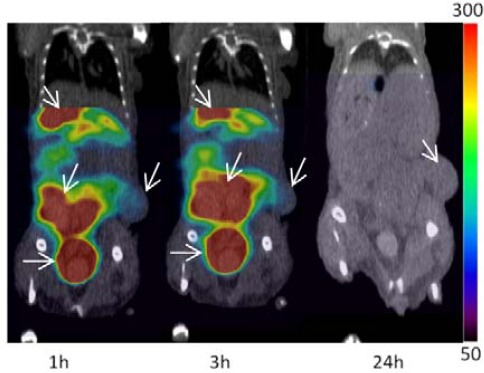
SPECT/CT image of [^125^I]PYK in U87MG.ΔEGFR xenograft at 1 hr, 3 hr, 24 hr post injection. The colon, liver, bladder and tumor were indicated by arrows. The thyroid was out of field of view in these SPECT images.

In nude mice (n=4) bearing xenografts of U87MG.ΔEGFR cells SPECT/CT imaging was performed at 1 hr, 3 hr and 24 hr post injection. High activity accumulations were visualized in the liver and colon at 1 hr and 3 hr, followed thereafter by decline, showing the clearance pathway of the [^125^I]PYK. Tumors could not be detected by [^125^I]PYK in 24 hr imaging (Figure 2).

## Discussion

Although gefitinib treatment is promising for many kinds of tumors depending on EGFR expression (13-15), its application as an effective therapy for GBM remains to be established (16). It was previously shown that NSCLC patients who had specific EGFR gene mutations, especially in-frame deletions in exon 19 (ΔLRE) and a point mutation in exon 21 (L858R), could benefit from gefitinib (17). These mutations, however, have rarely been reported in gliomas. The most frequent mutation in gliomas is the ΔEGFR mutation with the loss of exons 2 to 7 of the EGFR gene, resulting in an in-frame deletion of 267 amino acids in the extracellular domain (18). Although ΔEGFR is unable to bind to EGF, it is still constitutively phosphorylated and activates downstream signaling cascades, and promotes the survival and proliferation of glioblastoma cells (19). ΔEGFR may sensitize glioblastoma cells to EGFR kinase inhibitors by promoting chronic

dependence on phosphoinositide-3 kinase (PI3K) signaling. Some reports have shown the prognostic value of ΔEGFR mutation for TK inhibitor treatment and therefore suggested it as a target for cancer therapeutics (8, 20).

Previous studies have successfully shown through non-invasive imaging of EGFR expression, that the mutation status in NSCLC could aid in the selection of patients for individualized therapy with EGFR kinase inhibitors (21). However, the *in vivo* detection of EGFR expression and mutation in GBM, has been more difficult. Our study explored the use of [^125^I]PYK as a potential probe for detection of EGFR mutations in xenografts of U87 glioblastoma cells in mice to predict treatment with gefitinib treatment. In our previous study, we successfully imaged the EGFR mutation in NSCLC with [^125^I]PYK (10, 11). The biodistribution experiments demonstrated that the blood radioactivity was 0.80% of the injected dose per mL at 1hr and decreased to 0.03% of the injected dose per mL at 24 hr. Although the tumor uptake decreased from 4.37% of the injected dose per gram (%ID/g) at 1 hr to 1.53% of the injected dose per gram (%ID/g) at 24 hr, the tumor-to-blood radioactivity ratio increased at 24 hr after injection (10, 11). Based on these results, we examined the biodistribuion at 24 hr after the injection in the present study. We did not examine the stability of [^125^I]PYK *in vitro* nor measured thyroid uptake in mice after the injection of [^125^I]PYK. In the previous study, however, the uptake in the stomach at 1 hr post-injection was 6.10% of the injected dose per gram (%ID/g) and lower than in the liver of 27.0% of the injected dose per gram (%ID/g) and in the kidneys of 7.40% of the injected dose per gram (%ID/g). The uptake in the stomach decreased promptly. The thyroid could not be detected by SPECT imaging (10). Even if iodine may release from the compound, it is likely that de-iodination is not significant in this compound.

In gliomas over-expressing ΔEGFR with high phosphorylation activity, *in vivo* imaging showed no specific uptake in tumors. The differences in results of *in vivo* imaging in NSCLC and in gliomas might be due to the different genetic alterations of EGFR observed in gliomas although a previous study suggested that U87 cells were very sensitive to the EGFR inhibitor (22). The molecular mechanism determining the binding of 4-quinazoline compounds may be different as observed in NSCLC and therefore, needs to be more thoroughly investigated. In gliomas, clinical response requires sufficient plasma concentrations of EGFR tyrosine kinase inhibitor to abrogate downstream signaling (17). Also, cross-talk pathways of ΔEGFR and coactivation of EGFR TKs have been found frequently in GBMs (23), which might influence [^125^I]PYK specific binding to EGFR.

In conclusion, the failure of detection of EGFR expression in GBM, might be due to the complicated expression of EGFR status in GBM, suggesting that prediction of gefitinib effectiveness is difficult in GBM. As the activation and aberrant behavior downstream have been considered as new checkpoints for forecasting the results of TK inhibitor regimens, our further study will aim at developing radiolabeled agents for detecting the downstream pathway of EGFR.
